# New Anæsthetic Agent

**Published:** 1858-11

**Authors:** 


*New Anæsthetic Agent.—Acétone.*—We translate from the last number of Brown-Séquard's Journal of Physiology, a brief account of a new anæsthetic agent discovered by M. Béchamp. The experiments which have been made, appear to show that it is a discovery of great importance, and these observations, if confirmed, will place the newly discovered agent in advance of any anæsthetic now known. The following is the account from the French Journal:

M. Béchamp communicated to the Section some experiments which he had made in his laboratory, in the presence of several pupils, upon a new anæsthetic agent—acétone. This substance is much less disagreeable to breathe than amylene, its action is more rapid, but less persistent. It acts upon rabbits after thirty seconds; and the insensibility is such that one can mutilate the animal in any manner without pain. It is worthy of remark, that the prolonged inhalation of this agent had no fatal action on the rabbits.

